# Correlates of Undernutrition in Older People in Guadeloupe (French West Indies): Results from the KASADS Study

**DOI:** 10.3390/nu15132950

**Published:** 2023-06-29

**Authors:** Nadine Simo-Tabue, Denis Boucaud-Maitre, Laurys Letchimy, Jeff Guilhem-Decleon, Jeannie Helene-Pelage, Guillaume T. Duval, Maturin Tabue-Teguo

**Affiliations:** 1 Pôle Gériatrie-Gérontologie, CHU de Martinique, 97261 Fort-de-France, France; laurys.letchimy@chu-martinique.fr; 2 Centre Hospitalier le Vinatier, 69500 Bron, France; denis.boucaud@gmail.com; 3 Department of Geriatric Medicine, Angers University, CHU de Guadeloupe, 97110 Pointe-à-Pitre, France; jeff.gd@live.fr (J.G.-D.); jeannie.pelage@wanadoo.fr (J.H.-P.); 4 Department of Geriatric, FWI University, CHU d’Angers, 49100 Angers, France; guillaume.duval@chu-angers.fr; 5 Equipe EpiCliV, Université des Antilles, 34095 Montpellier, France; 6 Equipe ACTIVE, INSERM 1219, Université de Bordeaux, 33600 Pessac, France

**Keywords:** risk factors, undernutrition, aged, older adults, Guadeloupe

## Abstract

**Objectives**: This study aimed to determine the risk factors for undernutrition in community-dwelling older adults in Guadeloupe (Caribbean islands). **Methods**: We used data from the KArukera Study of Aging-Drugs Storage (KASADS), an observational cross-sectional study of community-dwelling older people living in Guadeloupe. The Mini Nutritional Assessment (MNA) was used to assess the risk of undernutrition. An MNA-short form (SF) score ≤11 defined the risk of undernutrition. Depression was assessed using the Center for Epidemiologic Studies Depression (CES-D) scale, cognitive function was assessed using the Mini Mental State Examination (MMSE), frailty was assessed using the Study of Osteoporotic Fractures index (SOF), and dependency was assessed using Lawton’s instrumental activities of daily living (IADL) scale. Bivariate and multivariate analyses were used to determine the correlates of undernutrition. **Results**: The study sample comprised 115 patients aged 65 years or older; 67.8% were women, and the mean age was 76 ± 7.8 years. The prevalence of undernutrition was 21.7% (95% CI = 15.2–30.1%). In our bivariate analysis, the risk of undernutrition was associated with MMSE score, IADL score, frailty, and CES-D score. We found no significant relation between nutrition risk and other variables, such as marital status, pain, or polypharmacy. In the multivariate analysis, the factors associated with the risk of undernutrition were MMSE score (Odd-Ratio (OR): 0.74 (0.58–0.97)) and CES-D score (OR: 1.13 (1.02–1.27)). **Conclusions**: Cognitive decline and the risk of depression were independently associated with the risk of undernutrition in community-dwelling older people in Guadeloupe. Although we cannot imply causality in this relation, the detection of these three key geriatric syndromes in community-dwelling elders is essential to prevent adverse health outcomes. Further studies are warranted to confirm these findings.

## 1. Introduction

Undernutrition is a frequent and serious geriatric syndrome that is a major contributor to vulnerability in older adults. Undernutrition is defined as a state resulting from deficiencies, excesses, or imbalances in a person’s food intake and expenditure of energy and/or nutrients. It leads to a reduction in lean and cellular mass [1]. Undernutrition is multifactorial, and reported risk factors include social isolation, financial precariousness, psychological factors, and socio-demographic status, among others [2]. It is known to be associated with various clinical conditions such as low body mass index (BMI), infection, cancer, diabetes, renal function, acute kidney injury, pulmonary disease, gastro-intestinal disorders, depression, cognitive or functional disorders, and comorbidities and polypharmacy [3]. Undernutrition is also associated with an increased risk of adverse health outcomes, including infection, impaired health-related quality of life (HRQoL), falls, longer length of stay when hospitalized, bedsores, and mortality [4,5]. The prevalence of undernutrition rises with increasing age [6], and in France, it ranges from 6.4% in rural areas to 18.5% in urban zones [4]. It is estimated that 32% of elderly people living at home suffer from malnutrition [6]. In France, new diagnostic criteria to measure undernutrition in the elderly were to be taken in consideration by the High Authority of Health and the French Nutrition Federation in 2019. In summary, to diagnose undernutrition, it is necessary to combine at least one phenotypic criterion (weight loss ≥ 5% in 1 month or ≥10% in 6 months or ≥10% compared with the usual weight before the onset of the disease; BMI < 22 kg/m^2^; confirmed sarcopenia) and an etiological criterion (reduction in food intake ≥50% for more than 1 week or any reduction in intake for more than 2 weeks compared with usual food intake and/or protein-energy requirements; reduced absorption (malabsorption/maldigestion), as well as a pathological situation (with or without inflammatory syndrome). Despite the fact that MNA, as well as its short form, MNA-SF (a validated tool to estimate nutritional status), allows us to identify the risk of undernutrition, it is not considered as a criterion to diagnose undernutrition. The MNA-SF comprises six questions relating to nutritional, functional, and cognitive status. It is quick and easy to administer, and shows a strong correlation and good agreement with the full-length MNA in older adults. The MNA-SF accurately identifies people at risk of and suffering from undernutrition in the community [7]. As with the majority of geriatric syndromes, people with undernutrition do not always receive the attention of primary care healthcare providers [8]. However, since 2019, the World Health Organization program for integrated care for older people (ICOPE), which aims to promote primary prevention initiatives, has highlighted nutritional status as an intrinsic capacity that is necessary for successful ageing [9,10]. By 2030, the population of Guadeloupe (a French territory in the Caribbean) is expected to decline. This is due to an ageing population. The proportion of people over 65 years old could account for 28% of the total population (INSEE projections). At the same time, there will be a considerable increase (the double) in the number of dependent elderly people. Studies reveal that this part of the population will have to face age-related medical problems and an increase in the prevalence of chronic pathologies (hypertension, diabetes, cancer) or neuropsychiatric diseases. Other consequences will be a growing number of avoidable hospitalizations. On the other hand, the island of Guadeloupe faces numerous challenges. Some diseases are prevalent and above the national (France) level with regards to ethnic and genetic characteristics. There are also specific social and economic characteristics. In addition to this, the geographical location of the island in terms of access to care is also challenging. In the current context, the economic, medical, social, and human stakes are considerable, and the question that needs to be addressed is to what extent does living in Guadeloupe have an impact on the care of the elderly population [11,12]. It is of particular importance to identify the factors associated with undernutrition in this context, especially since certain modifiable risk factors are susceptible to influence by the geographic, psycho-socio-cultural, and ethno-anthropological environment. To the best of our knowledge, to date, no study has investigated the risk factors for undernutrition in older adults living in the Caribbean islands. The aim of this study was to identify the correlates of undernutrition in older people in Guadeloupe.

## 2. Methods

### 2.1. Study Design

This observational, cross-sectional study in Guadeloupe, a French overseas department situated in the Caribbean sea, used data from the Karukera Study Aging-Drug Storage (KASADS) [11]. In the KASADS cohort, the selected patients were contacted over the phone by two medical school students. The two interviewers were trained in standardized geriatric assessment tools (falls, dementia, depression, undernutrition, confusion, psycho-social vulnerabilities, etc.) by the geriatrics team at the CHU de Guadeloupe. A total of 15 GPs from the region took part in recruiting participants (i.e., a maximum of 10 patients per GP). GPs systematically offered patients who met the inclusion criteria a chance to participate in the study until our quota of 8 patients was reached. The two investigators acquired verbal consent from all participants. The investigators visited the participants’ homes to collect the various parameters. Subjects who had not been correctly selected by the GPs were excluded upon home visitation.

All consenting participants received a study information leaflet explaining the study procedures, and all participants provided written informed consent.

The study was approved by the Ethics Committee of the University Hospital of Guadeloupe (A6_19_10_01_KASADS).

### 2.2. Data Recorded

The validated French-language version of the MNA-SF was used to assess the nutritional status of all patients [7]. Each participant was evaluated based on the following: food intake, weight loss, mobility, psychological stress or acute disease, neuropsychological problems (only dementia), and BMI. The final score was used to classify individuals as undernourished (0 to 7 points), at risk of undernutrition (8 to 11 points), or as having a normal nutritional status (13 to 14 points). For the purposes of this study, we considered all those with an MNA-SF score ≤11 to be at risk of undernutrition.

In the KASADS cohort, functional status was assess using Lawton’s instrumental activities of daily living (IADLs) [13] and Katz’s ADL scale [14]. Lawton’s IADL scale measures four dimensions: using the telephone, using transport, managing medications, and managing finances. Scores range from 0 to 4; a score of 0 indicates total autonomy, while a score of 4 indicates total dependency. Katz’s ADL scale measures 6 dimensions: bathing, toileting, transferring, eating, dressing, and continence. It appears that when there is an alteration of one or more ADLs, the elderly loses functional independence. The ADL provides a rating of each of the 6 items on a binary scale (0/1), with 1 indicating independence and 0 indicating dependency. The total score varies between 0 and 6. Cognitive function was assessed using the Mini Mental-State Examination [15]. This 30-item instrument evaluates cognitive function in terms of orientation, repetition, verbal recall, attention and calculation, language, and visual construction. Scores range from 0 to 30, and scores of 24/30 or lower indicate impaired cognitive function. Pain and HRQoL were evaluated using a visual analog scale (VAS). We also recorded socio-demographic characteristics (age, sex, level of education, marital status), usual alcohol intake (non-drinker, former drinker and current drinker) and tobacco consumption (non-smoker, former smoker current smoker), comorbidities (presence or absence of diabetes, hypertension, dyslipidemia), and BMI (calculated as the weight divided the square of the height in meters). We recorded all current medications, and polypharmacy was defined as the concomitant use of ≥5 different drugs per day [16].

Frailty syndrome was assessed using the Study of Osteoporotic Fractures index (SOF) [17], which comprises 3 items: involuntary weight loss of 5 kg or more, inability to rise from a chair 5 times without using the arms, and reduced energy level (answer “no” to the question “Do you feel full of energy?”). Meeting two or more criteria indicates frailty; 1/3 indicates pre-or-intermediate frailty; 0/3 indicates non-frail patients. Depression was evaluated using the Center for Epidemiologic Studies-Depression (CES-D) scale [18], which is a self-reported questionnaire comprising 20 items asking how often over the past week the person experienced symptoms associated with depression. Scores range from 0 to 60, with higher scores indicating greater depressive symptoms.

### 2.3. Statistical Analysis

Quantitative variables are expressed as mean ± standard deviation, and qualitative variables are expressed as either numbers or percentages. In our study population, no patient had an MNA-SF score <8, corresponding to undernutrition. Therefore, the variable MNA-SF was dichotomized, with individuals having a score > 11 considered not at risk and those with a score ≤ 11 considered at risk of undernutrition. Physical frailty (assessed by the SOF index scale) was studied by categorizing the SOF score into two groups: those with a score of 1 or 2 were considered frail, and those with a score of 0 were considered robust. Variables were compared between those at risk and those not at risk of undernutrition using the chi-square or Student’s *t*-test as appropriate. Variables at a 20% threshold in the univariate analysis were considered in a multivariable analysis using logistic regression. At last, the relation between MNA-SF and other variables was investigated in a multivariable analysis using a logistic regression model to identify the factors independently associated with risk of undernutrition. We excluded BMI score, considering that BMI is already part of the MNA assessment scale. No imputation was used to account for missing data. A *p*-value of <0.05 was considered statistically significant. All analyses were performed with RStudio software (v.3.0.2. 21).

## 3. Results

The study sample comprised 115 community-dwelling individuals aged 65 and older. The mean age of the participants was 76.0 ± 7.8 years; the mean BMI was 26.8 ± 5.3, and 67.8% were women. In total, 43.5% had diabetes, 87.0% had hypertension, 45.2% had dyslipidemia, and 21.7% (25/115 participants) were at risk of undernutrition. The mean HRQoL score was 66.2 ± 20.3, and for pain, the score was 51.6 ± 21.7. The mean IADL score was 3.4 ± 1.0, and the mean ADL score was 5.79 ± 0.83 (Table 1).

Table 1 presents the population characteristics according to nutritional status. On average, individuals at risk of undernutrition were older and more dependent. They were also more frequently pre-frail or frail compared to those with normal nutritional status (48.0% vs. 25.6%, respectively; *p* = 0.031). In bivariate analysis, those at risk of undernutrition more frequently were considered to be in cognitive decline (MMSE score 22.2 ± 3.8 vs. 24.1 ± 3.4; *p* = 0.015), have a higher CES-D score (13.8 ± 7.9 vs. 7.9 ± 6.1; *p* < 0.001), a lower BMI (22.3± 3.9 vs. 28.0 ± 5.1), and a lower IADL score (2.8 ± 1.4 vs. 3.6 ± 0.8). No significant relation was observed between the risk of undernutrition and the other variables, such as marital status, alcohol and tobacco consumption, pain, or polypharmacy. Table 2 shows the factors found to be significantly associated with the risk of undernutrition by multivariate analysis. The risk of undernutrition was associated with a low MMSE (*p* = 0.015) and CES-D score (*p* = 0.020). No significant association between age, IADL score, pain, physical frailty, alcohol consumption, and risk of undernutrition was found. We also did not observe any significant interaction effect between cognitive impairment and depressive symptoms on the risk of undernutrition (*p* = 0.741).

## 4. Discussion

In this study, cognitive decline assessed using the MMSE score and depressive symptoms (using the CES-D score) were both associated with an increased risk of undernutrition among community-dwellers aged 65 years and over in Guadeloupe. Our results are consistent with the data found in the literature on this subject. Different studies have already shown a correlation between cognitive impairment and risk of undernutrition. A study by an Italian pharmacovigilance group (Agencia Italiana del ARmaco) showed an association between cognitive disorders and certain markers of malnutrition, such as albuminemia and low BMI. In a prospective study with 32 years of follow-up, Stewart et al. reported that weight loss in participants preceded the onset of mild cognitive decline in the diagnostic trajectory of dementia [19,20,21]. This involuntary weight loss could be associated with both a loss of muscle mass and a loss of muscle strength, characteristics of sarcopenia, which seems to be more frequent in the elderly [22]. Several hypotheses have been proposed to explain undernutrition in people with cognitive disorders, particularly in people with Alzheimer’s disease [20]. Old people who suffer from cognitive decline have difficulty eating, shopping, or preparing meals. Atrophy of the mesial temporal cortex can lead to a loss of appetite [23]. Finally, other neurobiological factors have also been mentioned, such as a decrease in demanding hormones, such as neuropeptide Y or norepinephrine [24,25]. In our study, we also observed an association between depressive symptoms and undernutrition. This has also previously been described in studies performed in various contexts [26,27,28,29]. Weight loss, which is a contributing factor to MNA score, is listed as a possible symptom of depression in the Diagnostic and Statistical Manual of Mental Disorders 5th edition (DSM-5), apart from when it is attributable to a general condition. The possible explanations for this relate to the symptomatology of depression, which includes loss of appetite, general asthenia, and a loss of interest or pleasure in daily activities. However, a bi-directional relationship was also suggested by Lee et al. [26], whereby diseases that led to undernutrition could also be the cause of depressive symptoms. Depression is also thought to induce neuroendocrine modifications that disturb the regulation of food intake [29,30]. We observed a borderline significant link between pain and undernutrition (*p* = 0.06). Findings reported in the literature are conflicting with regard to this relationship, with some studies reporting that pain is a risk factor for undernutrition; however, few studies have specifically investigated this point. One Austrian study reported a link between severe pain and malnutrition risk in hospitalized patients [31]. Other studies have suggested a link between the intensity of pain and perceived alterations in appetite while accounting for Geriatric Depression Scale score, the number of painkillers one takes, and opioid use [32]. It has also been pointed out that side effects of painkillers, especially opiates, include reduced appetite, nausea, vomiting, or constipation [33]. Several elements could explain the lack of a statistically significant association in our study, notably the profile of the study population and the tools used to evaluate pain (a VAS) and depression (the CES-D instrument). Our data preclude any identification of the etiology or type of pain, which are key pieces of information guiding pain management. For example, cancer-related pain can be accompanied by hypercatabolism, which alone could account for undernutrition, whereas neuropathic pain or pain related to arthritis cannot. In our study, marital status was not found to be associated with nutritional status, although a meta-analysis published in 2020 including 16 cross-sectional studies showed an increased risk of malnutrition in those who were single, widowed, or divorced [2]. This could be due to a loss of pleasure in eating or poor dietary habits. A further explanation proposed was the lack of cooking experience in this generation of older men when they found themselves alone and having to cook for themselves. Given the lack of a statistical reason, there may be a cultural explanation for our findings. Indeed, the majority of studies to date that have reported a link between malnutrition and marital status were performed in western countries with populations who differed compared with ours, notably in terms of a culture of family solidarity and intergenerational interactions [12]. No link between nutritional status and polypharmacy was observed in our study. Again, the literature is discordant on this point. A meta-analysis of six longitudinal studies by Streicher et al. found no association with polypharmacy [34], while Zadak et al. underlined the difficulty of identifying a clear relationship between these two syndromes due to the abundance of confounding factors [35]. The lack of a significant relation in our study could be due to the different measurements used compared to other studies investigating polypharmacy and nutritional status. Indeed, contrary to other studies, we considered polypharmacy as the concomitant use of 5 or more drugs per day, whereas other studies considered hyper-polypharmacy, with a threshold at 10 drugs per day. In our study, alcohol consumption was also not found to be associated with nutritional status, which, although seemingly counter-intuitive, is congruent with previous reports. It is known that alcohol use can lead to deficiencies in micronutrients [36], but a risk of protein-energy malnutrition in older individuals has not been clearly described. In their review of the literature, van der Pols-Vijlbrief et al. included 28 observational studies of the risk factors for malnutrition, and no effect of alcohol was reported [37]. The proposed explanations include malabsorption, prolonged periods of fasting during hospitalization for complications of cirrhosis, and iatrogenic causes [36]. However, in our population, the majority of alcohol users reported only moderate consumption (one glass per day, with only three participants reporting an intake of more than three glasses of alcohol daily). Therefore, it is possible that only a very minute proportion would reach the stage of advanced liver disease. We could also hypothesize that, given the high average age of the patients in our study, any severe and chronic alcoholics would already have died since their life expectancy is shorter, leading to a potential survival bias [38].

Our results provide a good opportunity to underline the utility of performing systematic evaluations of depression risk and cognitive function during home visits and primary care consultations, either by GPs or nurses. Both depression and cognition can have a deleterious influence on nutritional status; therefore, early management is essential, especially considering that both are often underdiagnosed in older adults. In view of our findings and literature data, the MNA-SF scale appears to be an effective, user-friendly tool that would be easy to implement in routine practice. Furthermore, early intervention for depression or cognitive decline in older individuals before they begin to affect nutrition is an objective that is closely aligned with the practice and goals of GPs in primary care.

Our study has some limitations. The study design precluded the identification of any causal relationship between the risk of undernutrition on the one hand and depression or cognitive decline on the other hand. The few patients at risk of undernutrition limits the statistical power of the analyses. Nevertheless, despite a relatively small sample size (n = 115), the participants were representative of the general population of older individuals in the region in terms of comorbidities. Also, the MNA-SF is an instrument designed to capture the multidimensional nature of undernutrition. Our results are concordant with strategies designed to promote healthy nutritional status in older people, a key challenge and goal for GPs.

## 5. Conclusions

In this study of 115 community-dwelling individuals aged 65 years and older in Guadeloupe, we found that cognitive decline and depression were independently associated with the risk of undernutrition. Although our study design precludes concluding a causal relation, the detection of these three geriatric syndromes in older community-dwelling individuals is crucial for the prevention of adverse health outcomes. Further studies are required to confirm and expand on these findings.

## Figures and Tables

**Table 1:** Comparison of population characteristics according to risk of undernutrition.

	Total Sample n = 115	MNA-SF ≤ 11 (n = 25)	MNA-SF > 11 (n = 90)	*p*
Age, years—mean (±SD)	76.0 (±7.8)	78.9 ± 8.5	75.2 ± 7.5	0.040
Men	37 (32.2%)	8 (32.0%)	29 (32.2%)	0.983
BMI, Kg/m^2^—mean (±SD)	26.8 (±5.3)	22.3 ± 3.9	28.0 ± 5.1	<0.001
No diploma	39 (33.9%)	10 (40.0%)	29 (32.2%)	0.467
Lives alone	67 (58.3%)	16 (64.0%)	51 (56.7%)	0.511
Diabetes	50 (43.5%)	7 (28.0%)	43 (47.8%)	0.078
Hypertension	100 (87.0%)	23 (92.0%)	77 (85.6%)	0.397
Dyslipidemia	52 (45.2%)	10 (40.0%)	42 (46.7%)	0.553
Tobacco consumption	8 (7.0%)	1 (4.0%)	7 (7.8%)	0.511
Alcohol consumption	16 (13.9%)	1 (4.0%)	15 (16.7%)	0.105
Polypharmacy	72 (62.6%)	14 (56.0%)	58 (64.4%)	0.440
HRQoL/100—mean (±SD)	66.2 (±20.3)	61.8 ± 20.2	67.5 ± 20.3	0.217
Pain/100—mean (±SD)	51.6 (±21.7)	57.2 ± 27.6	50.0 ± 19.6	0.142
IADL/4—mean (±SD)	3.4 (±1.0)	2.8 ± 1.4	3.6 ± 0.8	<0.001
Frailty (SOF index)	35 (30.4%)	12 (48.0%)	23 (25.6%)	0.031
MMSE—mean (±SD)	23.7 (±3.6)	22.2 ± 3.8	24.1 ± 3.4	0.015
CES-D—mean (±SD)	9.2 (±7.0)	13.8 ± 7.9	7.9 ± 6.1	<0.001

SD: Standard deviation; BMI: Body Mass Index; HRQoL: Health-Related Quality of Life; IADL: Instrumental Activities of Daily Living; SOF, Study of Osteoporotic Fractures Index; MMSE: Mini Mental State Examination; MNA-SF: Mini Nutritional Assessment Short Form; CES-D: Center for Epidemiologic Studies Depression Scale.

**Table 2:** Factors associated with the risk of undernutrition by multivariate logistic regression.

Variable	Estimate	*p*	OR (95%CI)
Age	0.04	0.467	-
MMSE score	−0.29	0.015	0.74 (0.58–0.97)
Pain score	0.03	0.061	1.03 (1.00–1.06)
IADL score	0.43	0.313	-
Frailty (=yes)	0.03	0.808	-
CES-D score (depression)	0.13	0.020	1.13 (1.02–1.27)
Alcohol consumption (=yes)	−0.96	0.431	-

OR: odds ratio; IADL: Instrumental Activities of Daily Living; MMSE: Mini Mental State Examination; CES-D: Center for Epidemiologic Studies Depression Scale.

## Data Availability

The datasets used and/or analyzed during the current study are available from the corresponding author upon reasonable request.
